# Neuromodulation for post-stroke aphasia rehabilitation: established cortical stimulation and emerging network-based targets

**DOI:** 10.3389/fnhum.2026.1961051

**Published:** 2026-09-23

**Authors:** Mariagiovanna De Luca, Giovanni Federico, Daniela Rigatti, Onofrio Gigliotta, Paola Marangolo

**Affiliations:** 1 Department of Humanities Studies, University of Naples Federico II, Naples, Italy; 2 Department of Education, Psychology and Communication, Suor Orsola Benincasa University, Naples, Italy; 3 Institute of Cognitive Sciences and Technologies, National Research Council of Italy (CNR), Rome, Italy

**Keywords:** neuromodulation, photobiomodulation, post-stroke aphasia, repetitive transcranial magnetic stimulation, speech-language therapy, transcranial direct current stimulation

## Abstract

Post-stroke aphasia is a disabling disorder of language and communication for which speech-language therapy remains the standard of care. Because behavioural treatment yields variable gains and many people retain chronic communication limitations, non-invasive neuromodulation has been investigated to increase the responsiveness of residual language networks to therapy. This narrative review critically integrates evidence available through July 2026 for repetitive transcranial magnetic stimulation (rTMS), theta-burst stimulation, transcranial direct current stimulation (tDCS), and emerging approaches targeting the cerebellum, spinal sensorimotor pathways, and cellular metabolism. Recent meta-analyses support small-to-moderate average benefits of adjunctive cortical stimulation, especially for naming, but these estimates coexist with marked heterogeneity, small trials and incomplete evidence for generalisation, durability, functional communication, and quality of life. Recent randomised studies illustrate the value of pairing stimulation with a defined learning task and of using lesion, tract, functional-connectivity, or hemodynamic information to select targets and predict response. Cerebellar tDCS, transcutaneous spinal direct current stimulation, transcranial alternating current stimulation, and transcranial photobiomodulation are conceptually attractive but remain investigational because the clinical evidence is limited, inconsistent, or based on uncontrolled and proof-of-concept studies. Across modalities, serious adverse events are uncommon when contemporary screening and dosing guidance are followed, although stroke-specific comorbidity and seizure risk require trained supervision. Neuromodulation should therefore be viewed as a potentially useful, state-dependent amplifier of speech-language therapy rather than a replacement for it. Progress towards routine care will require adequately powered multisite trials, preregistered patient-level stratification, treatment-fidelity reporting, ecologically valid outcomes, longer follow-up, and direct comparisons of personalised with fixed protocols.

## 1 Introduction

Aphasia is an acquired impairment of language that most often follows damage to the language-dominant hemisphere after a stroke. It may affect speaking, comprehension, reading, writing, and pragmatic language use, with consequences extending far beyond impairment-level test scores. Approximately one third of people with acute stroke meet criteria for aphasia, although estimates depend on case definition and sampling (Engelter et al., 2006). Persistent aphasia limits autonomy, social participation, return to work, and access to health care, and is associated with reduced quality of life. Recovery is typically fastest during the first weeks and months, but meaningful treatment-related change remains possible years after stroke.

Speech-language therapy (SLT) is the foundation of aphasia rehabilitation. Systematic and randomised evidence supports structured, sufficiently intensive language intervention, including in chronic aphasia (Bhogal et al., 2003; Brady et al., 2016, 2025; Breitenstein et al., 2017). Nevertheless, the response is highly variable. Lesion location and volume, damage to connecting white-matter pathways, initial severity, time after stroke, domain-specific cognitive resources, treatment dose, and the fit between therapy and the impaired language process all shape outcome (Brady et al., 2025). Access to sustained high-dose treatment is also unequal (Brady et al., 2025). These realities have motivated interest in interventions that might increase experience-dependent plasticity during SLT (Davidson et al., 2024; Dawson et al., 2024).

Non-invasive neuromodulation alone does not restore language. Recent reviews have emphasised that non-invasive brain stimulation should be considered an adjunctive intervention designed to enhance the effects of evidence-based speech-language therapy rather than a stand-alone treatment (Harvey and Hamilton, 2022). Its clinically plausible role is to alter the physiological conditions under which a person practices language, thereby changing the probability, magnitude, or durability of learning. Repetitive transcranial magnetic stimulation (rTMS) and transcranial direct current stimulation (tDCS) have accumulated the largest evidence base (Lefaucheur et al., 2017; Elsner et al., 2019; Marangolo, 2020, 2025; Raymer and Johnson, 2024; Tan et al., 2024). More recent work has extended stimulation beyond conventional perisylvian targets to the cerebellum, primary motor and prefrontal cortices and spinal sensorimotor pathways (Marangolo, 2020, 2025). Transcranial photobiomodulation (tPBM) has additionally been proposed to influence cellular metabolism and neurovascular function (Naeser et al., 2020; Estrada-Rojas and Cedeño Ortiz, 2023).

Enthusiasm should be balanced against recurring limitations. Aphasia trials are often small, use heterogeneous interventions and outcomes, and preferentially report trained naming. A statistically significant improvement on practised items does not necessarily generalise to untrained words, connected speech, everyday communication, participation, or quality of life. Sham methods and masking also differ across devices, while crossover designs may be vulnerable to carryover when washout periods are short. In early post-stroke studies, spontaneous recovery and concurrent rehabilitation complicate causal inference. Finally, simple rules such as “excite the left hemisphere and inhibit the right” are increasingly difficult to reconcile with patient-specific network data.

This review critically updates the evidence through July 2026. We examine established cortical approaches and emerging network-based targets, distinguish therapeutic trials from mechanistic demonstrations, and organise the literature around three questions: what has been shown, what remains uncertain, and what evidence would be required for clinically responsible personalisation.

## 2 Methods of evidence identification and synthesis

We performed a structured narrative review of human research on neuromodulation for adult post-stroke aphasia. PubMed/MEDLINE was searched from database inception through 18 July 2026 using combinations of terms for stroke, aphasia, language rehabilitation, rTMS, theta-burst stimulation (TBS), tDCS, transcranial alternating current stimulation (tACS), cerebellar stimulation, transcutaneous spinal direct current stimulation (tsDCS), and photobiomodulation. Reference lists of relevant reviews and the full-text articles supplied with the manuscript were also screened. Priority was given to randomised or sham-controlled intervention studies, systematic reviews, meta-analyses, safety guidance, and mechanistic studies that directly informed target selection. Studies of primary progressive aphasia were excluded because the underlying neurodegenerative pathology and progressive course differ fundamentally from post-stroke aphasia, leading to distinct mechanisms of language impairment and potentially different responses to neuromodulation; mixed-aetiology samples were retained only when the post-stroke subgroup was identifiable or when the study illustrated a methodological issue. Protocols and non-peer-reviewed preprints were not used to support efficacy claims.

As this manuscript is a narrative review, the literature search and study selection were not conducted according to a formal systematic-review framework such as PRISMA. Accordingly, we did not perform a formal risk-of-bias assessment using standardized tools such as Cochrane RoB 2 or CASP. Instead, studies were critically appraised with respect to study design, sample characteristics, stimulation parameters, control conditions, outcome measures, and major methodological limitations. This approach was chosen to allow an integrative and critical discussion of the heterogeneous neuromodulation approaches and study designs represented in the aphasia literature. Because the objective of the review was interpretive rather than quantitative, we did not conduct a new meta-analysis.

## 3 A network and learning framework for aphasia neuromodulation

Language recovery after stroke reflects reconfiguration within a distributed, bilateral system rather than the behaviour of a single “language centre.” Indeed, longitudinal imaging has described an early reduction in language-network activity, followed by transient recruitment of bilateral or right-hemisphere regions and, in many patients, later re-engagement of residual left-hemisphere tissue (Saur, 2006; Hartwigsen and Saur, 2019). This group-level sequence is not a universal prescription. The contribution of the right hemisphere depends on which left-hemisphere regions are damaged rather than on lesion volume, premorbid lateralisation, time since stroke, task demands, and the remaining structural routes (Turkeltaub et al., 2026). Contemporary patient-level work demonstrates that right-hemisphere language-network recruitment can support performance in some people with aphasia rather than merely interfere with the damaged hemisphere (Raboyeau et al., 2008; Turkeltaub et al., 2026).

The influential interhemispheric-competition model proposed that loss of left-hemisphere output releases right-hemisphere homologues from transcallosal inhibition, after which excessive right-to-left inhibition impedes recovery (Rosen et al., 2000; Kiran, 2012). This model motivated inhibitory 1-Hz rTMS or cathodal tDCS over the right inferior frontal gyrus and excitatory stimulation of residual left-language cortex (Lefaucheur et al., 2017, 2020; Raymer and Johnson, 2024). Those strategies remain biologically plausible in selected patients, but the model should be regarded as one possible network state, not a default explanation for all aphasia. Suppressing the right hemisphere may be unhelpful when it is carrying compensatory language or domain-general control functions. Conversely, excitation of the structurally disconnected left cortex may not always engage the intended network.

The physiological labels “excitatory” and “inhibitory” also require caution. High-frequency rTMS and intermittent TBS often increase corticospinal excitability in the healthy motor cortex, whereas low-frequency rTMS and continuous TBS often reduce it (Tan et al., 2024). Similarly, anodal tDCS tends to depolarise and cathodal tDCS to hyperpolarise neuronal membranes in simplified preparations (Lefaucheur et al., 2017; Elsner et al., 2019; Marangolo, 2020, 2025). In the lesioned brain, however, response depends on orientation of the induced field, ongoing activity, local inhibitory-excitatory balance, medication, dose, prior stimulation, and network connectivity (Nitsche et al., 2003; Poreisz et al., 2007; Monte-Silva et al., 2013; Bikson et al., 2016). tDCS modulates the probability of firing rather than directly eliciting action potentials, and both electrodes contribute to the electric field (Nitsche and Paulus, 2000). Lesions and cerebrospinal fluid spaces can substantially distort current flow (Galletta et al., 2015). Polarity and frequency should therefore describe protocols, not predetermine their behavioural effects.

A learning framework offers a more clinically useful interpretation. Neuromodulation changes the state of a network while SLT specifies what is learned. The same stimulation may facilitate, impair, or leave behaviour unchanged depending on the task performed during the stimulation window (Marangolo, 2020; Shah-Basak et al., 2023). Pairing should be mechanistically coherent: phonological treatment should engage phonological computations, syntactic treatment should repeatedly recruit the relevant sentence network, and motor-speech practice should activate speech-motor circuitry (Marangolo, 2025). Dose must likewise be considered jointly; stimulation session number, treatment intensity, item exposure, and rest intervals are inseparable components of the intervention (Fregni et al., 2021; Marangolo, 2025). Table 1 summarises the principal neuromodulation approaches with respect to their primary physiological action, typical aphasia protocols, the current clinical evidence supporting their use, and the main unresolved questions.

**Table 1:** Neuromodulation modalities considered in post-stroke aphasia rehabilitation.

Modality	Primary physiological action	Typical aphasia protocol	Current clinical evidence	Principal uncertainty
rTMS	Magnetically induced cortical electric fields; repeated pulses alter local and network excitability.	Often 1 Hz over right pars triangularis or excitatory stimulation of residual left-language cortex; repeated sessions, preferably with SLT.	Most mature evidence; pooled small-to-moderate language effects, with important null trials.	Optimal target varies according to recovery phase and network state; adequacy of sham control, seizure screening, and functional generalization remain important considerations.
TBS	Patterned rTMS intended to induce plasticity in a shorter session.	Intermittent TBS to residual left cortex or continuous TBS to right homologues.	Positive pooled estimate, but single-session benefits can be transient.	Heterogeneous protocols and limited head-to-head evidence.
Cortical tDCS	Weak direct current changes membrane polarization and ongoing network responsiveness; both electrodes shape the field.	1–2 mA for about 20 min over 5–15 sessions during task-specific SLT.	Small average naming benefit; recent task-specific controlled studies are encouraging.	No universally superior montage; lesion-dependent current flow and limited functional outcomes.
Cerebellar tDCS	Modulation of cerebello-cortical prediction, timing, selection, and learning circuits.	Right posterolateral cerebellar anodal or cathodal stimulation during naming or other language therapy.	Mixed, small-sample results; occasional task-specific or qualitative functional communication effects.	Polarity cannot be mapped simply onto cortical excitation/inhibition; independent replication lacking.
tsDCS	Proposed modulation of ascending spinal, sensorimotor, and cerebello-thalamo-cortical pathways.	Thoracic stimulation during verb-focused therapy.	Verb-specific gains and connectivity changes in overlapping small cohorts.	No independent parallel-group replication; mechanism and durable functional relevance uncertain.
tACS	Sinusoidal current interacts with ongoing oscillatory timing and phase coupling.	Theta-range or 7-Hz stimulation during language or phonological-memory tasks.	Feasible proof-of-concept effects, sometimes only during stimulation.	Individual frequency, phase, entrainment persistence, and responder heterogeneity.
tPBM	Red/near-infrared light is proposed to affect mitochondrial metabolism and neurovascular function.	Scalp light-emitting devices used alone or during SLT.	Only uncontrolled case-series and single-case aphasia evidence.	Cortical dose is uncertain; no randomized sham-controlled efficacy trial.

SLT, speech-language therapy; rTMS, repetitive transcranial magnetic stimulation; TBS, theta-burst stimulation; tDCS, transcranial direct current stimulation; tsDCS, transcutaneous spinal direct current stimulation; tACS, transcranial alternating current stimulation; tPBM, transcranial photobiomodulation.

## 4 Transcranial magnetic stimulation

### 4.1 Mechanisms and canonical protocols

TMS uses a rapidly changing magnetic field to induce an electric field in the superficial cortex. A single pulse can transiently perturb or probe a cortical region; repeated pulses can alter excitability and network coupling beyond the stimulation period. Most aphasia studies have used 1-Hz rTMS over the right pars triangularis to reduce activity in a contralesional inferior-frontal target. Other studies have applied higher-frequency rTMS or intermittent TBS to residual left-hemisphere language cortex, continuous TBS to right-hemisphere cortex or targets selected from functional imaging (Tan et al., 2024).

The relative focality of TMS is an advantage for hypothesis-driven targeting, and neuronavigation can improve anatomical reproducibility. Disadvantages include cost, acoustic and somatosensory sensations that complicate masking, operator dependence, and a small seizure risk (Lefaucheur et al., 2020) TBS delivers patterned pulses in a shorter session and is attractive for rehabilitation scheduling, but short administration time does not remove the need for careful dose reporting or safety screening (Tan et al., 2024).

### 4.2 Therapeutic evidence

Early reports of right inferior-frontal 1-Hz rTMS described naming improvements in small chronic-aphasia cohorts, sometimes persisting for months (Naeser et al., 2005a, 2005b, 2010; Martin et al., 2009). These studies were important demonstrations but frequently lacked sham control and reused participants across reports, limiting independence and protection against practice effects. Subsequently, small, controlled studies reported benefits on naming or global aphasia measures (Barwood et al., 2011; Weiduschat et al., 2011), but the samples were heterogeneous and typically comprised only 10–12 participants. A crossover pilot suggested changes in selected discourse word counts, although direct active–sham differences were limited and multiple outcomes were tested (Medina et al., 2012). Later trials produced a still more nuanced picture. Heikkinen et al. (2019) paired right-hemisphere 1-Hz rTMS with intensive language-action therapy in chronic aphasia. Language therapy improved performance, but active stimulation did not add a reliable benefit over sham. Georgiou and Kambanaros (2022) similarly found variable responses to right pars triangularis 1-Hz rTMS and continuous TBS in a six-person single-subject series. Ilkhani et al. (2018) reported pre-post improvement after low-frequency rTMS, but the absence of a sham comparator prevents attribution to stimulation.

Other protocols have targeted the residual left cortex. Kakuda and collaborators (2010a) found that 1-Hz rTMS (20 min, 10 sessions over 6 days, followed by weekly sessions for 3 months) administered to the left Wernicke’s area in two patients with fluent aphasia resulted in a slight improvement in several subtests of the Standard Language Test of Aphasia. Similar results were obtained in another study by the same authors (Kakuda et al., 2010b) in which rTMS was applied to the left frontal lobe in two of four poststroke nonfluent patients. In the open-label study of Szaflarski et al. (2011) functional-MRI-guided intermittent TBS over the residual left Broca’s area improved semantic fluency in six of the eight chronic participants enrolled (*p* = 0.028), although the absence of a sham condition prevents attribution to stimulation. Cotelli et al. (2011) reported improved object naming in three chronic non-fluent patients who received high-frequency rTMS over the left dorsolateral prefrontal cortex immediately followed by 25 min of individualised speech therapy over 4 weeks; because comparable gains also followed placebo rTMS plus speech therapy, the improvement cannot be attributed to stimulation. A single session of intermittent TBS over the left posterior temporal region produced a short-lived auditory-comprehension benefit in a 13-person pilot study, evident minutes rather than weeks after stimulation (Versace et al., 2020).

Imaging studies show that stimulation can alter network activity, but physiological changes are not, in themselves, evidence of rehabilitation efficacy. For example, Winhuisen et al. (2005) used PET and disruptive rTMS to test acute right-hemisphere language involvement, and Xu et al. (2021) used functional MRI after a single session of left primary motor cortex intermittent TBS. Both were mechanistic studies, not treatment trials.

The strongest recent evidence comes from controlled adjunctive designs and quantitative synthesis. In a four-arm study of 40 people with Broca-type aphasia, the combination of rTMS and SLT outperformed either component alone, although the modest sample and limited methodological reporting constrain certainty (Yaşa et al., 2023). A 2026 double-blind randomised trial used functional near-infrared spectroscopy to identify a left-hemisphere language target before neuronavigated 10-Hz rTMS; 15 sessions combined with intensive SLT produced a greater naming gain than sham plus SLT in subacute aphasia (Li et al., 2026). The study is encouraging because target selection was biologically informed and the stimulation was embedded in therapy, but replication and functional outcomes are needed.

A 2026 meta-analysis of 26 randomised trials (419 active and 318 control participants) reported an overall language benefit of rTMS, with the largest subgroup estimate for inhibitory stimulation of the right pars triangularis and for subacute participants (Wei et al., 2026). A separate meta-analysis of 12 randomised TBS trials involving 524 participants also found a pooled benefit but substantial heterogeneity (Zhang et al., 2026). Network meta-analysis of 26 trials and 1,136 participants ranked some multimodal or bilateral combinations highly, yet certainty was moderate to high mainly for standard low-frequency rTMS-plus-SLT comparisons and low or very low for several top-ranked novel combinations because estimates were indirect and imprecise (Lin et al., 2026). Rankings should therefore not be mistaken for definitive head-to-head superiority.

Overall, rTMS has the most mature evidence among non-invasive stimulation approaches for aphasia, but its effects are not uniform, and guideline-level endorsement for routine aphasia treatment remains limited (Lefaucheur et al., 2020). The best-supported clinical research model is repeated stimulation paired with structured SLT, using prespecified targets and outcomes. Table 2 distinguishes representative therapeutic trials from mechanistic studies that are often conflated in narrative summaries.

**Table 2:** Representative TMS studies and evidence classifications in post-stroke aphasia.

Study; design	Target and stimulation	Behavioural treatment	Main finding	Critical interpretation, and level of evidence
Naeser et al. (2005a, 2005b); Case series; *n* = 4 chronic post-stroke aphasics	Right pars triangularis; 1 Hz; 1,200 pulses; 10 sessions.	None during study.	Naming improved in some participants and persisted for months.	Foundational but uncontrolled; later reports reused participants and are not independent replications. Level IV.
Winhuisen et al. (2005); Exploratory mechanistic crossover study, PET perturbation; *n* = 11 acute post-stroke PWA	PET-selected left and right IFG; brief 4-Hz trains during a semantic decision task. Single session.	None.	Disruption of right IFG impaired residual language in a subset.	Mechanistic evidence that right IFG can be compensatory, not a treatment trial. N/A.
Kakuda et al. (2010a); Case series; *n* = 2 chronic post-stroke PWA	Left Wernicke’s area; 1 Hz; 20 min; 10 inpatient sessions over 6 days, then weekly sessions for 3 months.	Language therapy throughout the inpatient and outpatient phases.	Token Test and selected Standard Language Test of Aphasia subtests improved and were maintained at 3 months.	Uncontrolled two-patient feasibility series with concurrent therapy; no sham comparator. Level IV.
Kakuda et al. (2010b); Case series; *n* = 4 chronic post-stroke PWA	fMRI-guided site homologous to the peak activation; 1 Hz; 1,200 pulses; 10 sessions (right frontal in two patients, left frontal in two).	Not reported as a controlled component.	Language function improved in all four patients.	Open case series; imaging-guided targeting is of interest, but there is no control condition. Level IV.
Barwood et al. (2011); Randomized sham-controlled trial, *n* = 12 chronic post-stroke PWA	Right pars triangularis; 1 Hz; 1,200 pulses; 10 sessions.	No concurrent SLT.	Active–sham differences on selected naming and repetition measures at 1 week.	Small cohort reported in several follow-up papers; publications should not be counted as independent trials. Level II.
Cotelli et al. (2011); Case series; *n* = 3 chronic post-stroke PWA	20-Hz rTMS over left DLPFC (BA 8/9), 90% RMT, 25 min/session, 5 sessions/week for 4 weeks (20 sessions), followed by 25 min of individualized speech therapy.	25 min of individualised speech therapy immediately after each session.	Object naming improved from 2 weeks and up to 48 weeks, after both real and placebo rTMS plus therapy.	Gains followed placebo as well as real stimulation, which is more consistent with a therapy than a stimulation effect. Level IV.
Szaflarski et al. (2011); Exploratory open-label study; *n* = 8 chronic post-stroke PWA	Residual left Broca’s area localised by fMRI; excitatory iTBS; 10 daily sessions.	None during the study.	Semantic fluency improved in six of the eight participants enrolled (*p* = 0.028), with a leftward shift in language lateralisation.	Uncontrolled and unblinded; requires sham-controlled replication. Level IV.
Weiduschat et al. (2011); Randomized controlled pilot study; *n* = 10 subacute post-stroke PWA	Right pars triangularis versus vertex; 1 Hz; 10 sessions.	Individualized SLT after stimulation.	Aachen Aphasia Test total improved more after IFG stimulation.	Encouraging pilot; heterogeneous syndromes and very small groups. Level II.
Medina et al. (2012); Randomized sham-controlled crossover study, *n* = 10 chronic post-stroke PWA	Individually selected right IFG site; 1 Hz; 10 sessions.	None.	Some discourse word counts increased at 2 months; direct active–sham evidence was limited.	Multiple uncorrected outcomes and complicated pooling reduce certainty. Level II.
Heikkinen et al. (2019); Randomized sham-controlled crossover study; *n* = 17 chronic post-stroke PWA	Right pars triangularis; 1 Hz; 20 sessions.	Naming during stimulation; intensive language-action therapy in weeks 3–4.	Both groups improved with therapy; no active-rTMS advantage or synergy.	Important null add-on trial. Level II.
Ilkhani et al. (2018); Single-arm before–after study; *n* = 24 chronic post-stroke PWA	Right Broca’s homologue; 1 Hz, single session.	None reported.	Verbal fluency scores improved from pre- to post-stimulation.	No sham comparator and no randomization; improvement cannot be attributed to rTMS. Level IV.
Versace et al. (2020); Exploratory randomized crossover study; *n* = 13 chronic post-stroke PWA	iTBS, left Wernicke’s area (CP5), 80% AMT, 600 pulses, 200 s; single session.	None.	Auditory comprehension improved 5 min after left stimulation, but not at 40 min.	Transient proof of concept, not rehabilitation efficacy. N/A.
Xu et al. (2021); Single-arm mechanistic pilot study; *n* = 16 post-stroke PWA	Left-M1 iTBS single session.	No therapy during stimulation/scan.	Immediate resting-state fMRI changes; no therapeutic language outcome was reported.	Mechanistic only. N/A.
Georgiou and Kambanaros (2022); Case series; *n* = 6 chronic post-stroke PWA	Right pars triangularis; either 1-Hz rTMS or cTBS; 10 sessions.	No concurrent SLT.	Variable individual language and quality-of-life changes.	Exploratory; no sham and no valid comparison between paradigms. Level IV.
Yaşa et al. (2023); Randomized sham-controlled study; *n* = 40 chronic post-stroke PWA	Right IFG; 1 Hz; 15 sessions.	SLT alone, rTMS alone, sequential combination, or no treatment.	Combination produced the broadest within-group gains.	No sham, baseline imbalance, ambiguous masking, multiple outcomes, and protocol inconsistencies. Level II.
Li et al. (2026); Randomized sham-controlled study; 28 enrolled, 27 completed (14 active, 13 sham); subacute post-stroke PWA	fNIRS-guided left-language target; neuronavigated 10-Hz rTMS; 15 sessions.	Intensive SLT in both groups.	Active stimulation produced a greater naming gain than sham.	Strong personalized-targeting signal; requires replication and functional outcomes. Level II.

PWA: persons with aphasia; cTBS, continuous theta-burst stimulation; RMT, resting motor threshold; IFG, inferior frontal gyrus; iTBS, intermittent theta-burst stimulation; M1, primary motor cortex; PET, positron emission tomography; SLT, speech-language therapy; TMS–EEG, transcranial magnetic stimulation–electroencephalography; Level II: randomized controlled trial; Level IV: Case series/Single arm pre-post; N/A, not applicable.

## 5 Cortical transcranial direct current stimulation

### 5.1 Mechanisms, dosing, and targeting

tDCS applies a weak direct current between scalp electrodes. Conventional sponge-electrode montages are inexpensive, portable, and easy to combine with therapy, but their electric fields are diffuse. High-definition arrangements can improve model focus, although clinical superiority remains unproven. Typical aphasia protocols deliver 1–2 mA for approximately 20 min over 5–15 sessions while participants practice language. Left anodal, right cathodal, and bihemispheric montages have all been used; montage names alone are insufficient because electrode size, return-electrode location, lesion anatomy, and current direction determine the field (Lefaucheur et al., 2017, 2020; Esmaeilpour et al., 2018; Marangolo, 2025).

Unlike TMS, conventional tDCS does not directly evoke measurable neuronal responses but instead modulates ongoing neuronal excitability (Marangolo, 2020, 2025). Consequently, its effects are strongly influenced by the functional state of the stimulated network and are therefore typically evaluated in conjunction with behavioural therapy (Marangolo, 2020, 2025).

The interaction between neuromodulation and behavioural therapy is likely to depend not only on the stimulated cortical target but also on the specific neural processes engaged by the task performed during stimulation. Speech-language therapy provides a behaviourally induced state of network activation that may influence how TMS or tDCS modulates cortical excitability and activity-dependent plasticity. Rather than acting independently of ongoing neural processing, the effects of neuromodulation appear to depend, at least in part, on the functional state of the stimulated network and on the cognitive processes engaged during stimulation (Stagg and Nitsche, 2011; Fertonani and Miniussi, 2017). Thus, intensive and repetitive language practice may preferentially engage the residual neural circuits supporting the language trained function, providing a substrate on which neuromodulation can act.

This state-dependent interaction may be particularly relevant when the behavioural intervention is designed to repeatedly recruit a specific language or cognitive-linguistic process. For example, therapies targeting verb retrieval and thematic role processing, such as Verb Network Strengthening Treatment (VNeST), may repeatedly engage left inferior frontal and distributed language networks; importantly, studies combining VNeST with anodal tDCS over the left inferior frontal gyrus have provided preliminary evidence that stimulation delivered during this training may enhance treatment-related changes (Matar et al., 2022; Sheppard et al., 2025). In reading-based rehabilitation, iReadMore provides a particularly clear example of this principle: intensive reading practice engages distributed orthographic, phonological, semantic, frontal and occipital components of the reading network, while concurrent anodal tDCS over the left inferior frontal gyrus produced a small additional benefit on word-reading accuracy (Woodhead et al., 2018; Kerry et al., 2019). These examples suggest that the behavioural intervention should not be considered merely as an adjunct to stimulation, but as an important determinant of the neural state in which stimulation is delivered.

From this perspective, the therapeutic effect of neuromodulation may depend on the convergence between the stimulation-induced modulation, the residual structural and functional language network, and the language processes repeatedly engaged during therapy. Behavioural interventions that primarily target lexical-semantic retrieval, phonological processing, reading, action-verb processing, or sentence production may therefore provide different opportunities for state-dependent neuromodulation. Conversely, the same behavioural intervention may engage different residual networks across patients because of lesion location, network disconnection, and individual patterns of functional reorganisation. This interaction may contribute to the heterogeneity of neuromodulation outcomes observed across aphasia studies and supports the need to consider both the target of stimulation and the behavioural state induced during treatment when designing and interpreting combined neuromodulation–SLT interventions.

### 5.2 Evidence for language outcomes

Since word-finding difficulties are the most common symptom observed in aphasia, the majority of aphasia studies published through July 2026 have used a picture-naming task in combination with tDCS (Baker et al., 2010; Fiori et al., 2011; Flöel et al., 2011; Fridriksson et al., 2011, 2018; Kang et al., 2011; Marangolo et al., 2013a; Vestito et al., 2014; Shah-Basak et al., 2015; Wu et al., 2015; Norise et al., 2017; Silva et al., 2018; Rezaei et al., 2023; Alemanno et al., 2024; Bonilha et al., 2024; Ilardi et al., 2026). More specifically, there were 13 studies focused only on noun naming (Baker et al., 2010; Fiori et al., 2011; Flöel et al., 2011; Fridriksson et al., 2011, 2018; Kang et al., 2011; Shah-Basak et al., 2015; Wu et al., 2015; Norise et al., 2017; Silva et al., 2018; Rezaei et al., 2023; Alemanno et al., 2024; Bonilha et al., 2024) three studies on verb naming (Marangolo et al., 2013a; Fiori et al., 2019; Ilardi et al., 2026) and 2 in which both a noun and verb naming treatment approach was used (Fiori et al., 2013; Vestito et al., 2014) (see Table 3). Although picture naming has not been considered as an evidence-based treatment for aphasia, it has dominated the tDCS literature because it is readily standardised and repeatedly trained. It has been favoured because it promotes repetition and treatment intensity, thereby providing a constrained and replicable training method (Marangolo, 2025). Very few studies have combined tDCS with a theoretically driven treatment for verb production, such as the Verb Network Strengthening Treatment (VNeST) aimed to improve verb retrieval within different language contexts (Galletta and Vogel-Eyny, 2015), and ACTION, a cognitive protocol focused on verb inflexion and sentence construction (De Aguiar et al., 2015; Matar et al., 2022).

**Table 3:** Selected cortical tDCS studies informing the current evidence base.

Study; design	Montage and dose	Behavioural treatment	Main finding	Critical interpretation and level of evidence
Baker et al. (2010); Randomized sham-controlled crossover study; *n* = 10 chronic post-stroke PWA	Individualized anodal tDCS over left perilesional frontal cortex identified by fMRI during overt naming; 1 mA; 20 min/session; 5 sessions combined with computerized anomia treatment (picture naming of nouns).	Noun picture naming.	Anodal tDCS significantly improved naming accuracy for trained items compared with sham; improvements were maintained at 1-week follow-up. Patients with preserved perilesional cortex closer to the stimulation site showed the greatest benefit.	Provides early evidence that individualized stimulation of preserved left-hemisphere language regions can enhance lexical retrieval; however, the small sample, crossover design, and limited generalization beyond trained items restrict conclusions about clinical efficacy. Level II.
Fiori et al. (2011); Randomized sham-controlled crossover study; *n* = 3 chronic post-stroke PWA	Anodal tDCS over left posterior temporal cortex (Wernicke’s area); 1 mA; 20 min/session; 5 consecutive daily sessions combined with intensive picture naming training.	Noun picture naming.	Anodal tDCS significantly improved word retrieval accuracy and reduced naming latencies compared with sham; benefits persisted at follow-up (1–3 weeks after treatment).	Provides early evidence that left temporal facilitation can enhance lexical retrieval during naming therapy; however, the very small sample and exploratory design limit conclusions about efficacy and generalizability. Level II.
Flöel et al. (2011); Randomized sham-controlled crossover study; *n* = 12 chronic post-stroke PWA	Anodal, cathodal, or sham tDCS over right temporo-parietal cortex; 1 mA; 20 min/session; combined with short-term high-frequency anomia training (picture naming).	Noun picture naming.	Anodal tDCS over the right temporo-parietal cortex significantly enhanced the training effect compared with sham with gains maintained for at least 2 weeks. Baseline naming ability predicted responsiveness to anodal stimulation.	Provides evidence that facilitation of contralesional temporo-parietal regions can augment lexical retrieval training in chronic aphasia; however, the small sample, short intervention period, and heterogeneous response pattern limit conclusions about long-term clinical efficacy. Level II.
Fridriksson et al. (2011); Randomized sham-controlled crossover study; *n* = 8 chronic post-stroke PWA	Individualized anodal tDCS over left perilesional cortex identified by fMRI during picture naming; cathode over contralateral forehead; 1 mA; 20 min/session; 10 sessions of computerized anomia treatment (5 active, 5 sham).	Noun picture naming.	Anodal tDCS combined with naming therapy significantly reduced reaction time during picture naming for trained items, with effects maintained at 3-week follow-up.	Provides evidence that individualized fMRI-guided stimulation of preserved left-hemisphere regions can enhance lexical access efficiency; however, the small sample, task-specific effects, and limited generalization restrict conclusions about broader language recovery. Level II.
Jung et al. (2011); Single-arm before-after study; *n* = 30 chronic post-stroke PWA	Anodal tDCS over left perilesional language cortex; 2 mA; 30 min/session; 10 sessions combined with individualized speech-language therapy.	Melodic intonation therapy, visual action therapy, and auditory comprehension training.	Active tDCS combined with individualized language therapy produced greater improvements in overall aphasia severity (Aphasia Quotient) compared with behavioural therapy alone; benefits were influenced by aphasia type and baseline severity.	Suggests that tDCS may enhance recovery when paired with tailored behavioural interventions across different aphasia domains; however, the heterogeneous therapy protocols make it difficult to determine which language treatments specifically benefit from stimulation. Level IV.
Kang et al. (2011); Randomized sham-controlled crossover study; *n* = 10 chronic post-stroke PWA	Cathodal tDCS over right Broca’s homologue (right inferior frontal cortex) with anodal reference electrode over left supraorbital region; 2 mA; 20 min/session; 5 consecutive daily sessions combined with picture naming.	Noun picture naming.	Cathodal tDCS over the right Broca’s homologue significantly improved picture naming accuracy compared with sham, with effects observed immediately after the stimulation period.	Supports the interhemispheric inhibition model, suggesting that reducing maladaptive right frontal overactivity may facilitate lexical retrieval after left-hemisphere stroke; however, the small sample, short treatment duration, and lack of long-term follow-up limit conclusions about sustained efficacy. Level II.
Marangolo et al. (2011); Randomized sham-controlled crossover study; *n* = 3 chronic post-stroke PWA with speech apraxia/ articulatory deficits	Anodal tDCS over left inferior frontal gyrus (Broca’s area); 1 mA; 20 min; 5 consecutive daily sessions combined with intensive articulatory training and repetition tasks.	Non word repetition.	Anodal tDCS produced greater improvement in articulatory accuracy than sham, with generalization to other oral language tasks (word repetition and reading) and effects maintained at 1-week, 1-month, and 2-month follow-ups.	Provides initial evidence that left IFG stimulation may enhance recovery of speech motor planning/articulatory gestures; however, the extremely small sample and exploratory crossover design require confirmation in larger controlled studies. Level II.
Vines et al. (2011); Randomized sham-controlled crossover study; *n* = 6 chronic post-stroke PWA	Anodal tDCS over right posterior inferior frontal gyrus (right Broca’s homologue); 1 mA; 20 min; 3 consecutive daily sessions combined with Melodic Intonation Therapy (MIT).	Melodic Intonation Therapy.	Anodal tDCS combined with MIT produced greater improvements in speech fluency compared with sham-tDCS + MIT, supporting enhanced recruitment of right-hemisphere sensorimotor networks involved in melodic and articulatory speech production.	Provides preliminary evidence that tDCS may augment MIT-induced recovery by facilitating compensatory right-hemisphere mechanisms; however, the very small sample, short stimulation period, and crossover design limit conclusions about clinical efficacy and long-term effects. Level II.
You et al. (2011); Randomized controlled study; *n* = 21 subacute post-stroke PWA	Anodal tDCS over left superior temporal gyrus (Wernicke’s area) or cathodal tDCS over right Wernicke’s homologue; 2 mA; 30 min/session; 10 sessions combined with conventional speech-language therapy.	Conventional speech and language therapy.	All groups improved in overall aphasia severity, spontaneous speech, and auditory comprehension; cathodal tDCS over the right Wernicke’s homologue produced greater gains in auditory verbal comprehension than anodal left temporal or sham stimulation.	Supports the interhemispheric inhibition model, suggesting that reducing maladaptive right-hemisphere overactivity may facilitate comprehension recovery in early post-stroke aphasia; however, the subacute stage, heterogeneous global aphasia sample, and lack of long-term follow-up limit generalization. Level II.
Fiori et al. (2013); Randomized sham-controlled crossover study; *n* = 7 chronic post-stroke PWA	Anodal tDCS over left frontal cortex (Broca’s area) vs. left temporal cortex (Wernicke’s area) vs. sham; 1 mA; 20 min; 5 consecutive daily sessions combined with intensive naming training.	Noun and verb picture naming.	Verb naming improved significantly after anodal tDCS over the left frontal region, with effects persisting at 1-month follow-up; temporal stimulation preferentially benefited noun retrieval.	Demonstrates domain- and region-specific effects of tDCS, suggesting that left frontal stimulation may preferentially enhance verb retrieval mechanisms; however, the very small sample and within-subject design limit generalization. Level II.
Marangolo et al. (2013a); Randomized sham-controlled crossover study; *n* = 7 chronic post-stroke PWA	Anodal tDCS over left Broca’s area or left Wernicke’s area; sham control; 1 mA; 20 min/session; 5 consecutive daily sessions combined with intensive verb naming training.	Verb naming.	Anodal tDCS over left Broca’s area significantly enhanced verb retrieval accuracy compared with temporal stimulation and sham; improvements persisted at 1-month follow-up.	Provides evidence for functional specificity of left frontal regions in verb retrieval and supports targeted tDCS combined with intensive language therapy; however, the very small sample and crossover design limit generalizability and require replication in larger trials. Level II.
Marangolo et al. (2013b); Randomized sham-controlled crossover study; *n* = 12 chronic post-stroke PWA	Anodal tDCS over left Broca’s area or left Wernicke’s area; 1 mA; 20 min/session; 10 daily sessions combined with intensive Conversational Therapy.	Conversational therapy.	Anodal tDCS over left Broca’s area produced greater improvements in informative speech, with increased Content Units, verbs, and sentence production compared with Wernicke’s stimulation and sham; effects generalized to untrained discourse material and persisted at 1-month follow-up.	Demonstrates that tDCS can enhance ecological speech production when paired with conversational therapy, with a stronger effect of frontal stimulation on discourse generation; however, the small sample and crossover design limit generalization and require confirmation in larger trials. Level II.
Marangolo et al. (2013c); Randomized sham-controlled crossover study; *n* = 8 chronic post-stroke PWA with apraxia of speech	Bihemispheric tDCS: anode over left Broca’s area and cathode over right Broca’s homologue; 2 mA; 20 min/session; 10 sessions combined with intensive speech therapy.	Non word repetition.	Active bihemispheric tDCS significantly improved speech articulation and repetition accuracy compared with sham, with effects maintained after treatment.	Provides preliminary evidence that bihemispheric modulation of inferior frontal regions may enhance recovery of speech motor planning/articulatory processes; however, the small sample and crossover design limit conclusions about efficacy and generalizability. Level II.
Marangolo et al. (2014); Randomized sham-controlled crossover study; *n* = 8 chronic post-stroke PWA	Anodal tDCS over left Broca’s area or left Wernicke’s area; 1 mA; 20 min/session; 10 daily sessions combined with intensive Conversational Therapy.	Conversational therapy.	Stimulation over left Broca’s area produced greater improvements in spontaneous speech, including increased Content Units, verbs, sentences, and use of connective words to enhance linguistic cohesion; effects generalized to untrained discourse tasks and persisted at 1-month follow-up.	Demonstrates that tDCS combined with conversational therapy can enhance ecological discourse production beyond single-word retrieval; however, the small sample and crossover design limit generalization and require replication in larger controlled trials. Level II.
Vestito et al. (2014); Pilot longitudinal sham-controlled study; *n* = 3 chronic post-stroke PWA	Anodal tDCS over left dorsolateral prefrontal cortex (DLPFC); 2 mA; 20 min/session; 10 sessions combined with individualized language therapy.	Noun and Verb picture naming.	Active tDCS combined with language treatment improved naming performance for nouns and verbs, with gains maintained for up to 3 months after stimulation.	Provides preliminary evidence for long-lasting facilitation of lexical retrieval after tDCS, but the extremely small sample and pilot uncontrolled nature limit conclusions regarding efficacy and generalizability. Level II.
Campana et al. (2015); Randomized sham-controlled crossover study with VLSM analysis; *n* = 20 chronic post-stroke PWA	Anodal tDCS over left inferior frontal gyrus; 2 mA; 20 min/session; 10 sessions combined with language therapy.	Conversational therapy.	Active tDCS significantly improved picture description, noun naming, and verb naming compared with sham. VLSM revealed that lesions involving basal ganglia, insula, and superior/inferior longitudinal fasciculi predicted reduced responsiveness to stimulation.	Demonstrates that tDCS efficacy depends on the structural integrity of residual left-hemisphere language networks; highlights the importance of lesion anatomy and white-matter connectivity for patient selection and individualized neuromodulation approaches. Level II.
de Aguiar et al. (2015); Randomized sham-controlled crossover study; *n* = 9 chronic post-stroke PWA	Individualized tDCS targeting left perilesional frontal or temporal areas (MRI-guided); 1 mA; 20 min/session; 10 daily sessions combined with ACTION (verb inflection and sentence construction therapy).	Verb treatment “ACTION.”	ACTION improved production of treated and untreated verbs; active tDCS enhanced verb retrieval and promoted generalization beyond trained items, although the magnitude of stimulation effects varied across participants.	Provides evidence that tDCS may enhance linguistically motivated verb therapy and facilitate generalization; however, the small sample, crossover design, and possible sham ceiling effects limit conclusions about efficacy. Level II.
Shah-Basak et al. (2015); Randomized sham-controlled pilot study; *n* = 12 chronic post-stroke PWA	Individualized tDCS targeting left perilesional language cortex or right homologous regions; anodal or cathodal stimulation according to individual response; 2 mA; 20 min/session; 5 sessions combined with individualized language therapy	Noun picture naming.	Individualized active tDCS produced greater improvements in naming performance than sham, with benefits maintained at follow-up; responders showed heterogeneous patterns depending on stimulation site and polarity.	Supports personalized tDCS approaches based on individual functional anatomy and responsiveness; however, the small sample, exploratory design, and heterogeneous montages limit conclusions about optimal targeting and clinical efficacy. Level II.
Wu et al. (2015); Randomized sham-controlled crossover study; *n* = 12 chronic post-stroke PWA	Anodal tDCS over left posterior perisylvian region; 2 mA; 20 min; 20 sessions combined with speech-language therapy.	Noun picture naming.	Active tDCS significantly improved picture naming and auditory word–picture identification compared with sham; EEG measures suggested increased activation of left-hemisphere language networks after stimulation.	Supports the role of left posterior perisylvian stimulation in enhancing lexical retrieval when combined with language therapy; however, the small sample, crossover design, and absence of long-term follow-up limit conclusions about sustained clinical efficacy. Level II.
Marangolo et al. (2016); Randomized sham-controlled crossover study; *n* = 9 chronic post-stroke PWA with articulatory disorders	Bihemispheric tDCS: anodal stimulation over left Broca’s area and cathodal stimulation over right Broca’s homologue; 1 mA; 20 min; 15 sessions combined with intensive articulatory language therapy.	Non word Repetition.	Active tDCS improved articulation accuracy for treated stimuli and generalized to untreated language tasks; resting-state fMRI showed increased functional connectivity within left-hemisphere language networks after stimulation.	Supports bihemispheric modulation as a strategy to enhance articulatory recovery and promote left-hemisphere network reorganization; however, the small sample, crossover design, and preliminary neuroimaging findings limit conclusions about efficacy and mechanisms. Level II.
Norise et al. (2017); Randomized sham-controlled pilot study; *n* = 12 chronic post-stroke PWA	Anodal tDCS over left inferior frontal gyrus (Broca’s area); cathode over right supraorbital region; 2 mA; 20 min/session; 10 sessions combined with language tasks	Noun picture naming.	Active tDCS improved aspects of language production, with greater gains in sentence productivity and lexical selection in participants with poorer baseline performance; naming outcomes showed task-specific variability.	Suggests that baseline severity and task demands influence responsiveness to tDCS; however, the small pilot sample and heterogeneous language outcomes limit conclusions about specific effects on noun retrieval. Level II.
Silva et al. (2018); Randomized sham-controlled crossover study; *n* = 14 chronic post-stroke PWA	Bilateral tDCS: anode over left supraorbital region and cathode over right Broca’s homologue; 2 mA; 20 min; 5 consecutive sessions combined with naming tasks.	Noun picture Naming.	Active tDCS improved naming performance compared with sham, particularly response time and use of naming strategies, although accuracy effects were limited.	Provides preliminary evidence that short-term bilateral tDCS may facilitate naming performance; however, the small sample, brief stimulation protocol, and limited behavioural outcomes restrict conclusions about clinical efficacy. Level II.
Woodhead et al. (2018); Randomized crossover study; *n* = 21 chronic post-stroke PWA with central alexia	Anodal tDCS over left inferior frontal gyrus; 1 mA; 20 min; 11 sessions delivered concurrently with iReadMore training (4-week blocks; ~34 h therapy).	iReadMore training.	iReadMore significantly improved single-word reading accuracy for trained words; concurrent anodal tDCS produced a small additional facilitation of learning and promoted generalization to untrained words, but no significant improvement was observed for sentence or text reading.	Demonstrates that tDCS may enhance intensive computer-based reading rehabilitation and facilitate transfer beyond trained items; however, effects were modest, limited to word-level reading. Level II.
Feil et al. (2019); Randomized sham-controlled study; *n* = 12 postacute post-stroke PWA	Bihemispheric tDCS: anode over left IFG and cathode over right IFG; 2 mA; 20 min; 10 daily sessions combined with individualized language therapy.	Personalized speech and language therapy (not specified).	Active tDCS produced greater improvements than sham in picture naming and communication measures, with effects maintained at 1-month follow-up; neuroimaging suggested a leftward shift of language-related activity.	Supports the interhemispheric modulation model and the potential of bihemispheric tDCS as an adjunct to individualized language therapy; however, the small sample size and postacute heterogeneous population limit generalizability. Level II.
Fiori et al. (2019); Randomized sham-controlled crossover study; *n* = 20 chronic post-stroke PWA	Cathodal HD-tDCS over right Broca’s homologue; 1 vs. 2 mA; 20 min/session; 5 consecutive daily sessions combined with verb naming task.	Verb picture naming.	Cathodal HD-tDCS at 2 mA significantly improved verb naming, with effects maintained 1 week after treatment; no benefit was observed with 1 mA stimulation.	Demonstrates that HD-tDCS can enhance verb recovery through focal modulation of contralesional inhibitory activity, but highlights the critical role of current intensity/density; the short-term follow-up limit conclusions on clinical efficacy. Level II.
Fridriksson et al. (2018); Phase-II randomized controlled study; *n* = 74 chronic post-stroke PWA	Individualized anodal stimulation over preserved left temporal cortex; 1 mA; 20 min/session; 15 sessions.	Computerized naming therapy in both groups.	Adjusted active–sham difference favoured active by 5.7 names, but the confidence interval crossed zero.	Supported continued study rather than definitive efficacy. Level II.
Vila-Nova et al. (2019); Randomized sham-controlled crossover study; *n* = 12 chronic post-stroke PWA	Anodal tDCS over left Broca’s area; cathode over right supraorbital region; 2 mA; 20 min; single-session offline stimulation.	Syllable repetition and word production.	Active tDCS significantly improved articulatory accuracy during naming compared with sham; trends towards better word production and syllable repetition did not reach statistical significance.	Provides preliminary evidence that left IFG stimulation may enhance speech motor-linguistic processes, but the small sample, single-session design, and absence of intensive language training limit conclusions regarding sustained therapeutic effects. Level II.
Duncan and Nakkawita (2020); Randomized sham-controlled crossover study; *n* = 10 chronic post-stroke PWA (4 completed the full protocol)	Anodal tDCS over left inferior frontal gyrus (Broca’s area); cathode over right forehead; 2 mA; first 20 min of each 60-min speech-language therapy session; 16 sessions over two 8-week treatment phases.	Storytelling task.	Active tDCS combined with standard aphasia therapy showed a large effect favouring active stimulation on discourse production measured through Correct Information Units (CIUs) during Cinderella storytelling.	Provides preliminary evidence that tDCS may enhance ecological discourse outcomes when paired with usual language therapy; however, the very small sample, feasibility design, and crossover limitations prevent conclusions about efficacy. Level II.
Guillouët et al. (2020); Multicentric randomized sham-controlled crossover study; *n* = 10 post-stroke PWA (acute/subacute and chronic stages)	Bihemispheric tDCS combined with individualized speech-language therapy; 2 mA; 20 min; 15 sessions over 3 weeks with 1-week washout.	Personalized speech and language therapy (not specified).	No significant advantage of active tDCS over sham for spontaneous speech measures (number of different nouns, verbs, utterances, grammatical errors, or paraphasias) or cognitive-linguistic outcomes.	Suggests that bihemispheric tDCS combined with standard speech-language therapy may not enhance ecological measures of spontaneous language in a short treatment window; small sample, mixed aphasia stages, and complex outcome measures limit interpretation. Level II.
Ihara et al. (2020); Randomized sham-controlled crossover study; *n* = 6 chronic post-stroke PWA	Anodal tDCS over left inferior frontal cortex (Broca’s area; F5); cathode over right orbitofrontal cortex; 1.5 mA; 20 min; intermittent sessions combined with spoken word–picture matching (deblocking) and subsequent naming/sentence production training.	Word-picture matching task.	tDCS during the word–picture matching task enhanced subsequent naming accuracy and improved production of related words and sentences, including generalization to untrained items compared with sham.	Provides preliminary evidence that stimulating semantic retrieval processes before impaired language production may enhance therapy-induced gains; however, the very small sample, within-subject design, and short-term outcomes limit conclusions regarding clinical efficacy and durability. Level II.
Cherney et al. (2021); Randomized controlled pilot study; *n* = 20 chronic post-stroke PWA	Anodal tDCS over individualized left perilesional cortex (fMRI-guided); 1 mA; 13 min; 30 sessions combined with ORLA.	Sentence reading (ORLA) + production of functional language.	Active tDCS combined with ORLA yielded greater improvements in sentence reading and functional language production than ORLA with sham stimulation.	Supports the feasibility of pairing intensive reading-based therapy with individualized neuromodulation, although the pilot sample size and individualized targeting limit the generalizability of the findings and warrant confirmation in larger RCTs. Level II.
Pisano et al. (2021); Randomized sham-controlled crossover study; *n* = 14 chronic post-stroke PWA with severe agraphia	Dual tDCS: anode over left temporo-parietal cortex, cathode over right temporo-parietal cortex; 2 mA; 20 min; 10 sessions combined with writing training.	Nonwords writing.	Active dual tDCS significantly improved writing accuracy compared with sham, with generalization to untreated language tasks.	Provides the first controlled evidence supporting dual temporo-parietal tDCS as an adjunct to writing rehabilitation, although the small sample, crossover design, and focus on severe agraphia limit generalizability. Level II.
Zhao et al. (2021); Randomized sham-controlled study; *n* = 18 chronic post-stroke PWA	Anodal stimulation over left IFG; cathode over right deltoid; 2 mA; 20 min; 20 sessions combined with speech-language therapy.	Personalized speech and language therapy (not specified).	Active tDCS combined with speech-language therapy produced greater improvements in Aphasia Quotient and expressive language (spontaneous speech, repetition, and naming) than sham. Comprehension did not differ between groups.	Supports left IFG anodal tDCS as an adjunct to conventional speech-language therapy for nonfluent aphasia, but the small sample size, single-center design, and lack of long-term follow-up limit confidence in the generalizability and durability of the effects. Level II.
Matar et al. (2022); Randomized sham-controlled study; *n* = 6 chronic post-stroke PWA	Anodal stimulation over left IFG; 2 mA; 20 min; once weekly for 6 weeks combined with Verb Network Strengthening Treatment (VNeST).	Semantic treatment to improve verb retrieval.	Active tDCS combined with VNeST showed larger improvements in discourse production and functional communication than sham, with excellent feasibility and tolerability.	Preliminary findings support the feasibility of combining semantic verb therapy with tDCS, but the very small sample and feasibility design preclude conclusions about clinical efficacy and require confirmation in adequately powered RCTs. Level II.
Rezaei et al. (2023); Randomized sham-controlled crossover study; *n* = 16 chronic post-stroke PWA	Individually selected IFG or STG anodal/cathodal montage; 1 mA; 5 sessions.	No paired SLT.	Active stimulation improved repeated-item naming accuracy and aphasia score.	Montage optimization and exclusion of nonresponders before randomization create selection and regression-to-the-mean bias. Level II.
Yan et al. (2023); Randomized controlled three-arm study; *n* = 39 chronic post-stroke PWA	Anodal tDCS combined with Melodic Intonation Therapy; 2 mA; 20 min/session; 5 sessions/week for 3 weeks.	Melodic Intonation Therapy.	MIT combined with active tDCS produced greater improvements in language (WAB-AQ) and cognition than MIT with sham or conventional therapy.	Suggests additive benefits of pairing behavioural therapy with neuromodulation, although the small single-center sample and short follow-up limit conclusions about long-term efficacy and generalizability. Level II.
Alemanno et al. (2024); Case series; *n* = 4 chronic post-stroke PWA	Right inferior-frontal anodal stimulation; 1.5 mA; 20 sessions.	Computerized naming.	Interhemispheric connectivity increased; behavioural naming changes were inconsistent.	Imaging target engagement without robust clinical benefit. Level IV.
Bonilha et al. (2024); *Post-hoc* subgroup reanalysis; *n* = 70 chronic post-stroke PWA	Same trial and protocol as Fridriksson et al. (2018).	Same naming treatment.	Broca subgroup showed a larger 4-week active–sham difference and a marginal subtype interaction.	Hypothesis-generating; not an independent RCT and not prospectively powered for subtype. N/A.
Rubi-Fessen et al. (2024); Case series; *n* = 37 early postacute post-stroke PWA	Imaging-informed left anodal target; 2 mA; 10 sessions after 10 SLT-only sessions.	Same individualized SLT across periods.	Corrected gains were larger during the later SLT-plus-tDCS period.	Stimulation is inseparable from treatment order, cumulative therapy, practice, and spontaneous recovery. Level IV.
Khairina et al. (2025); Nonrandomized controlled pilot study; *n* = 30 chronic post-stroke PWA	Left IFG anode/right supraorbital cathode; 2 mA; 5 sessions.	Multidomain language training in both groups.	Writing was the only significant between-group domain; most comparisons were null.	No sham or randomization, short follow-up, and no multiplicity correction. Level III-2.
Twardochleb et al. (2025); Nonrandomized comparative study; *n* = 90 mixed etiology	Left prefrontal (F3) anode/right prefrontal (F4) cathode; 1–1.5 mA; sessions of 30–40 min across 5 months.	tDCS, SLT, or combination.	All groups improved; the combined group improved most (FAST +32.6%), exceeding both tDCS alone (+24.3%) and SLT alone (+17.8%).	No sham; mixed stroke/TBI cohort; unequal contact and no stroke-only analysis. Level III-2.
Gilardone et al. (2026); Randomized sham-controlled crossover study; *n* = 8 chronic post-stroke PWA	Left BA6 (anode) – contralateral supraorbital area (cathode); 1.5 mA, 20 min/session, daily for 4 weeks (20 sessions).	Sentence-to-picture matching and Mapping Therapy.	Both conditions improved; active stimulation yielded larger adjusted comprehension gains and CETI improvement.	Promising generalization beyond naming, but very small crossover sample. Level II.
Ilardi et al. (2026); Randomized sham-controlled crossover study; *n* = 32 chronic post-stroke PWA	Left IFG anodal tDCS; 2 mA; 10 active and 10 sham sessions.	Verb-naming therapy in both conditions.	Active stimulation enhanced verb naming relative to sham through 1 month.	Task-specific positive trial; lesion and tract integrity predicted response. Level II.

PWA, persons with aphasia; BA6, Brodmann area 6; CETI, Communicative Effectiveness Index; IFG, inferior frontal gyrus; SLT, speech-language therapy; STG, superior temporal gyrus; TBI, traumatic brain injury; tDCS, transcranial direct current stimulation; VLSM, Voxel-Lesion-Symptom Mapping; Level II: randomized controlled trial; Level III-2: non-randomized controlled/comparative study with concurrent controls; Level IV: Case series/Single arm pre-post.

Independently of the electrode configurations (uni vs. bihemispheric), type of stimulation (left anodal tDCS vs. right cathodal tDCS) and stimulation intensity (1 mA vs. 2 mA) (see Table 3), all of these studies have pointed to an additional effect of tDCS on noun and verb naming within a multiple session paradigm (Baker et al., 2010; Fiori et al., 2011, 2019; Flöel et al., 2011; Fridriksson et al., 2011, 2018; Kang et al., 2011; Marangolo et al., 2013a; Vestito et al., 2014; Shah-Basak et al., 2015; Wu et al., 2015; Norise et al., 2017; Silva et al., 2018; Rezaei et al., 2023; Alemanno et al., 2024; Bonilha et al., 2024; Ilardi et al., 2026). Importantly, in some of these studies tDCS effects were still present at 1 to 4 weeks (Baker et al., 2010; Fiori et al., 2011, 2013; Flöel et al., 2011; Fridriksson et al., 2011; Marangolo et al., 2013a; Shah-Basak et al., 2015; Norise et al., 2017; Silva et al., 2018) up to 2 to 6 months (Vestito et al., 2014; Shah-Basak et al., 2015; Norise et al., 2017; Silva et al., 2018) post-intervention, thus, revealing a persistence of the language improvement due to tDCS over time. Beyond improvements in picture naming, Zhao et al. (2021) also reported that anodal tDCS combined with speech-language therapy produced greater language recovery than behavioural treatment alone in individuals with nonfluent post-stroke aphasia, further supporting the role of tDCS as an adjunctive intervention rather than a stand-alone treatment (see Table 3).

More recently, the phase-II randomised futility trial by Fridriksson et al. (2018) was designed to determine whether anodal tDCS over the preserved left temporal cortex, combined with computerised naming therapy, showed sufficient promise to justify further clinical evaluation. Although the active stimulation group improved by approximately six additional correctly named items compared with sham (adjusted between-group difference 5.7 words, 95% CI − 0.9 to 12.3), the confidence interval included the possibility of no effect. Consequently, the findings did not provide definitive evidence of efficacy but supported continued investigation in larger, adequately powered clinical trials. A later diagnostic-subgroup analysis suggested a greater benefit in participants with Broca aphasia, illustrating both the promise and the risks of *post-hoc* stratification (Bonilha et al., 2024).

Recent trials broaden the picture (Rezaei et al., 2023) compared four stimulation configurations in a small crossover study and found improved naming following individually selected inferior frontal or superior temporal stimulation. However, the findings should be interpreted cautiously given the limited sample size (*n* = 16). Alemanno et al. (2024) observed connectivity changes following right-anodal tDCS in 4 participants without a corresponding robust naming effect, underscoring that a neural biomarker cannot substitute for clinical benefit. Rubi-Fessen et al. (2024) reported larger gains during a period of individualised stimulation plus SLT than during an earlier period of SLT alone in early post-acute aphasia; because treatment order was fixed and recovery was ongoing, the effects of stimulation, cumulative therapy, and time could not be separated. Khairina et al. (2026) found mostly nonsignificant between-group differences in a quasi-experimental sample of 30 participants. Twardochleb et al. (2025) reported the greatest improvement in a combined tDCS-and-SLT group within a 90-person multicenter comparative study, but mixed stroke/traumatic etiologies, lack of sham, and non-equivalent treatment conditions limit aphasia-specific causal inference.

More rigorous and task-specific work is encouraging. Ilardi et al. (2026) randomised 32 people with chronic aphasia in a double-blind crossover study of 10 sessions of left inferior-frontal anodal versus sham tDCS during verb therapy. Active stimulation produced larger verb-naming gains that persisted for 1 month. Preserved anterior tissue and inferred tract integrity predicted response, supporting network-based stratification (Ilardi et al., 2026).

The application of tDCS in aphasia has also been extended beyond remediation of word-finding difficulties to the recovery of speech production (Marangolo et al., 2013b, 2014; Campana et al., 2015), articulatory difficulties (Marangolo et al., 2011, 2013c, 2016; Vila-Nova et al., 2019), sentence comprehension (Gilardone et al., 2026), word writing (Pisano et al., 2021) and reading (Woodhead et al., 2018; Cherney et al., 2021) (see Table 3).

Some studies have combined anodal tDCS over the LIFG with Conversational Therapy (Marangolo et al., 2013b, 2014; Campana et al., 2015). Similarly, Guillouët et al. (2020) demonstrated that combining tDCS with speech-language therapy significantly improved spontaneous speech production compared with sham stimulation, supporting the ecological value of neuromodulation beyond single-word naming tasks. Compared to the sham condition, the combined effects of tDCS with conversational therapy resulted in the production of more informative speech, which persisted at 1 month after the end of the treatment and extended to materials that had been administered at the beginning and end of the therapy sessions (Marangolo et al., 2013b, 2014; Campana et al., 2015). Similarly, in a study by Duncan and Nakkawita (2020), a small sample of four nonfluent PWA benefitted from a tDCS protocol combined with a storytelling task, which enhanced their ability to produce more informative content units compared to the sham condition (Duncan and Nakkawita, 2020). In a smaller double-blind crossover study, (Gilardone et al., 2026) paired left premotor anodal tDCS with 4 weeks of sentence-comprehension treatment in eight people with chronic nonfluent agrammatic aphasia. Both conditions improved, but active stimulation yielded larger model-adjusted comprehension gains and improvement on a functional communication measure. The four-month washout reduced, but did not eliminate, the interpretive limitations of a very small crossover sample. These findings are consistent with earlier evidence reported by You et al. (2011), who showed that cathodal tDCS applied over the right Wernicke’s homologue improved auditory comprehension in individuals with subacute post-stroke aphasia, suggesting that neuromodulation may facilitate language recovery beyond lexical retrieval.

Six studies demonstrated tDCS efficacy in the recovery of articulatory deficits when combined with nonword and word repetition (Marangolo et al., 2011, 2013c, 2016; Vila-Nova et al., 2019) or with Melodic Intonation Therapy (Vines et al., 2011; Cipollari et al., 2015; Yan et al., 2023) (see Table 3). Very recent studies have also demonstrated tDCS’s effectiveness in treating severe agraphia (Pisano et al., 2021) and central alexia (Woodhead et al., 2018; Cherney et al., 2021).

Meta-analytic estimates are positive but modest. A 2020 network meta-analysis concluded that some tDCS configurations may improve naming, while emphasising small trials, low certainty, and uncertainty across montages (Elsner et al., 2020). A 2026 meta-analysis of 12 randomised trials and 400 participants estimated a small naming effect overall (standardised mean difference 0.25, 95% confidence interval 0.05–0.45) and a larger estimate in chronic aphasia, but outcomes and protocols remained heterogeneous (Wang et al., 2026). Such pooled results support a signal, not a universal protocol.

Three conclusions follow. First, active tDCS should be tested as an addition to an evidence-based treatment for aphasia, with sham participants receiving the same therapy dose. Second, trained-item naming is a proximal endpoint; trials also need untrained items, discourse and functional communication outcomes, and persistency of the achieved gains. Third, apparent subgroup effects require prospective validation (Marangolo, 2020, 2025). Selected recent studies are summarised in Table 3.

## 6 Emerging network-based targets and modalities

### 6.1 Sensorimotor and domain-general cortical targets

Language production depends on sensorimotor and executive systems as well as classical perisylvian cortex (Gili et al., 2017; Hertrich et al., 2021; Shamma et al., 2021). Primary motor cortex can influence articulatory planning and action-language networks, while dorsolateral prefrontal cortex may support working memory, response selection, and cognitive control (Nagel et al., 2008; Tomaiuolo et al., 2021; Butler et al., 2023). These targets could be useful when therapy explicitly recruits their computations. However, evidence that modulation of a domain-general node improves everyday communication remains sparse, and improvements may reflect attention or response selection rather than the recovery of a language representation. Future studies should specify the predicted mediator and include non-language control measures to test whether effects are language specific.

### 6.2 Cerebellar stimulation

The posterolateral cerebellum participates in language prediction, timing, response selection, and error-based learning through crossed connections with cerebral association cortex (Macher et al., 2014; Mariën et al., 2014; Yuan et al., 2022). It is often structurally spared after a middle cerebral artery stroke, making it an accessible node from which to influence a damaged cerebral network. Polarity effects in the cerebellum are particularly difficult to infer, and protocols have used both anodal and cathodal stimulation (Grimaldi et al., 2014; Manto et al., 2022).

In a randomised crossover study of 12 people with chronic aphasia, right cerebellar cathodal tDCS enhanced verb generation but not verb naming, suggesting task specificity rather than a generalised lexical effect (Marangolo et al., 2018). Kim et al. (2024) used a double-blind within-subject design with 28 participants across anodal and cathodal cohorts and found improvement in qualitative dimensions of functional communication after cerebellar tDCS plus computerised treatment, but not in communication independence. Zheng et al. (2025) found no added Western Aphasia Battery language benefit in 22 actively treated participants with anodal tDCS of the right cerebellum paired with speech therapy compared with a nonconcurrent historical sham group, although some quality-of-life domains improved. The historical comparator weakens causal inference.

More recent evidence remains mixed. Xu et al. (2021) randomised 16 participants to cathodal cerebellar tDCS or sham with SLT. Unadjusted between-group differences were not significant; a baseline-adjusted analysis favoured active stimulation, and functional near-infrared spectroscopy showed connectivity changes. Carthery-Goulart et al. (2026) studied six Cantonese-speaking participants in a crossover design and found high variability without a consistent active-stimulation advantage. A secondary analysis of a 19-person crossover cohort found that integrity of cerebello-cortical white-matter pathways predicted naming response (Johnson et al., 2026). This biomarker result is valuable for mechanistic and stratification purposes, but does not, by itself, establish average efficacy.

Cerebellar tDCS is therefore a rational experimental adjunct, particularly for tasks involving response selection, sequencing, or error-based learning. Current samples are too small and outcomes too inconsistent for routine use. Trials should use concurrent randomised sham controls, lesion-aware tract measures, and therapy tasks chosen to engage the targeted cerebral-cerebellar circuit.

### 6.3 Transcutaneous spinal direct current stimulation

tsDCS applies direct current over the spinal cord and is proposed to influence ascending sensorimotor and cerebello-thalamo-cortical pathways. This “bottom-up” route is attractive because it avoids direct stimulation of the damaged cortex. In a 14-person crossover study, five sessions of thoracic anodal or cathodal tsDCS were compared with sham during language treatment; active stimulation selectively improved trained verb production at short follow-up (Marangolo et al., 2017). A subsequent functional connectivity study in 16 participants found anodal tDCS-related improvements in verb performance and changes in a cerebello-cortical network (Marangolo, 2020). Fourteen participants overlapped with the earlier cohort, so the reports are not independent replications. The verb specificity is mechanistically interesting, but efficacy remains unconfirmed in a parallel-group trial.

### 6.4 Transcranial alternating current stimulation

tACS delivers sinusoidal current to interact with ongoing neural oscillations. It offers a direct test of temporal coordination in language networks but raises difficult questions about individual frequency, phase alignment, current spread, and whether entrainment persists after stimulation (Grover et al., 2023). In a 13-person proof-of-concept study, 7-Hz high-definition tACS paired with language treatment yielded better production and stronger entrainment in an in-phase condition than sham (Keator et al., 2026). A separate 13-person study targeting theta-frequency activity reported a transient phonological short-term-memory benefit during stimulation, with marked responder heterogeneity and no durable group effect (Erickson et al., 2026). These studies support feasibility and mechanism; they do not establish rehabilitation efficacy. Replication should preregister frequency selection, masking assessment, phase condition, and delayed functional outcomes.

### 6.5 Transcranial photobiomodulation

Transcranial photobiomodulation (tPBM) delivers red or near-infrared light to the scalp. Proposed mechanisms include modulation of cytochrome-c oxidase, cellular energy metabolism, cerebral blood flow, oxidative stress, and inflammation. Most mechanistic evidence is indirect, and the dose reaching the human cortex varies with wavelength, power, scalp and skull properties, and device geometry.

Clinical aphasia evidence is preliminary. Naeser et al. (2020) reported on six people with chronic post-stroke aphasia treated across four sequential, nonrandomized light-emitting diode montage cohorts. Naming and functional connectivity changed in some configurations, but the design cannot separate treatment, practice, and montage effects. Estrada-Rojas and Cedeño Ortiz (2023) described one person who improved more during 5 months of tPBM plus SLT than during the preceding 5 months of SLT alone. The sequential single-case design is vulnerable to cumulative treatment, time, and expectancy effects. TPBM should currently be described as experimental; randomised, dosimetrically transparent, sham-controlled trials are required before claims of clinical benefit.

Table 4 summarises emerging approaches and their principal evidential constraints.

**Table 4:** Emerging network-based stimulation approaches in post-stroke aphasia.

Study; design	Montage and dose	Behavioural training	Main finding	Critical interpretation and level of evidence
Marangolo et al. (2018); cerebellar tDCS. Randomized sham-controlled crossover study; *n* = 12 chronic post-stroke PWA	Cathodal right cerebellum (1 cm below and 4 cm lateral to the inion); reference electrode on the right shoulder; 2 mA, 20 min/session; 5 daily sessions per condition.	Verb generation and verb naming.	Benefit for verb generation, not verb naming.	Task-specific signal in a small sample. Level II.
Kim et al. (2024); cerebellar tDCS. Randomized sham-controlled crossover study; *n* = 28 chronic post-stroke PWA	Right cerebellum, (1 cm below and 4 cm lateral to the inion); reference electrode on the right shoulder; 2 mA, 20 min/session; 15 sessions of active tDCS and 15 sham sessions, 3–5 sessions/week, with 2-month washout.	Computerized lexical-semantic treatment.	Qualitative communication improved through 2 months; communication independence was null.	Primary crossover trial; cohort overlaps with Johnson et al. (2026); small absolute proxy-rated effect. Level II.
Zheng et al. (2025); cerebellar tDCS. Randomized sham-controlled crossover study; active *n* = 22 vs. sham *n* = 25 chronic post-stroke PWA.	Anodal right cerebellum (1 cm below and 4 cm lateral to the inion); cathode on right deltoid; 2 mA, 20 min/session; 5 consecutive days.	Personalized SLT.	No language advantage; selected quality-of-life domains improved.	Nonconcurrent groups differed substantially in chronicity; multiple secondary outcomes. Level II.
Xu et al. (2025); cerebellar tDCS. Randomized controlled study; 16 recruited/14 analysed chronic post-stroke PWA.	Cathodal right cerebellum (1 cm below and 4 cm lateral to the occipital tuberosity); anode on right shoulder; 2 mA, 20 min/session; 10 consecutive days.	Individualized SLT.	Primary unadjusted between-group language comparison was null; adjusted estimate and connectivity findings favoured active.	Very small and heterogeneous; no delayed outcome. Level II.
Carthery-Goulart et al. (2026); cerebellar tDCS. Randomized sham-controlled crossover study; *n* = 6 chronic post-stroke PWA.	Right posterolateral cerebellum; anode at PO10 (2 mA); two return electrodes at P10 and O10 (−1 mA each); 2 mA, 20 min/session; 5 sessions.	Computerized noun/verb naming.	No active naming benefit; verb accuracy improved after sham in a *post-hoc* contrast.	Null proof of concept with high variability. Level II.
Johnson et al. (2026); cerebellar tDCS. Exploratory biomarker study; *n* = 19 chronic post-stroke PWA.	Right cerebellum (1 cm below and 4 cm lateral to inion); reference on right shoulder; 2 mA, 20 min/session; 15 active + 15 sham sessions, 3–5/week; 2-month washout.	Naming treatment.	Cerebello-cortical white-matter integrity predicted naming response.	Predictive mechanism; does not establish average efficacy. N/A.
Marangolo et al. (2017), Marangolo (2020); tsDCS. Randomized sham-controlled crossover study; *n* = 14 and *n* = 16 chronic post-stroke PWA.	Anodal thoracic spinal stimulation, active electrode over T10; reference electrode on the upper limb/shoulder region; 2 mA, 20 min/session; 5 consecutive daily sessions per condition; anodal, cathodal and sham conditions.	Verb-focused language treatment.	Verb-specific gains and cerebello-cortical connectivity changes.	Fourteen participants overlapped: not independent replications. Level II.
Keator et al. (2026); HD-tACS. Exploratory controlled study; *n* = 13 chronic post-stroke PWA	Two 2 × 1 HD ring montages targeting left IFG pars opercularis and posterior MTG/STG; frontal center at F5 with surrounding F7/FC5; posterior center at CP5 with P5/TP7; 1 mA, 7 Hz, 25 min stimulation blocks; in-phase, anti-phase and sham; 3 sessions, ≥48 h apart.	Language treatment.	In-phase stimulation increased words produced and oscillatory entrainment versus sham.	Mechanistic small-sample evidence; durability and functional impact unknown. N/A.
Erickson et al. (2026); theta-tACS. Exploratory controlled study; *n* = 13 chronic post-stroke PWA	Two 2 × 1 HD montages targeting left inferior frontal and temporoparietal regions; frontal center F5 surrounded by F7/FC5; TP center CP5 surrounded by P5/TP7; 2 mA total, 4–7 Hz, 20 min/session; in-phase, anti-phase and sham; 3 sessions.	Phonological short-term-memory task.	Transient benefit during stimulation; no durable group effect and marked responder heterogeneity.	Not rehabilitation efficacy. N/A.
Naeser et al. (2020); tPBM. Case series; *n* = 6 chronic post-stroke PWA	LED placements varied across four protocols: bilateral/midline SMA; ipsilesional left hemisphere; ipsilesional LH + mPFC; ipsilesional LH + mPFC + precuneus; 500 mW LED cluster, red 633 nm + NIR 870 nm, 22.2 mW/cm^2^; 18 treatments, 3/week for 6 weeks.	No controlled SLT comparator.	Some montages were associated with naming and connectivity changes.	No sham/randomization; dose and montage changed together. Level IV.
Estrada-Rojas and Cedeño Ortiz (2023); tPBM. Single case post-stroke PWA	First LED cluster applied to 8 left-hemisphere language targets; subsequently an 810-nm LED helmet over the whole scalp; LED cluster: 630/660/850 nm, 200 mW/cm^2^, 12 J/cm^2^/min; helmet: 810 nm, 24 mW/cm^2^, 28.8 J/cm^2^; 45-min sessions, 2/week for 5 months (30 sessions).	SLT during second phase.	Descriptive speech-language improvement was larger in the combined phase.	Sequential design cannot separate stimulation, cumulative therapy, time, or expectancy. Level IV.

PWA, persons with aphasia; IFG, inferior frontal gyrus; MTG/STG, middle temporal gyrus/superior temporal gyrus; LED, light-emitting diode; SLT, speech-language therapy; tACS, transcranial alternating current stimulation; tDCS, transcranial direct current stimulation; tPBM, transcranial photobiomodulation; tsDCS, transcutaneous spinal direct current stimulation. Level II: randomized controlled trial; Level IV: Case series/Single arm pre-post.

## 7 Safety, tolerability, and implementation

When delivered within contemporary guidance, non-invasive stimulation is generally well tolerated. Common tDCS and tACS effects include tingling, itching, warmth, transient erythema, headache, fatigue, or light flashes. Serious adverse events have not shown a causal signal within conventional low-intensity tDCS parameters, but reporting quality varies (Bikson et al., 2016; Antal et al., 2026). TMS commonly causes scalp discomfort, facial-muscle contraction, headache, and acoustic exposure. Seizure is rare but is the major acute risk, especially with higher-frequency protocols and in patients with predisposing factors (Rossi et al., 2021).

Stroke populations require more than a generic safety checklist. Screening should address seizure history, unstable medical conditions, cranial metal or implanted electronic devices, skull defects, medications that alter seizure threshold, pain and sensory impairment that may affect tolerability reporting, and cognitive or language limitations that affect consent. Hearing protection is required for TMS. Device-specific emergency procedures and trained supervision should be documented. Adverse events and withdrawals should be reported by randomised condition rather than described only as “well tolerated.”

Masking should also be treated as an outcome. Scalp sensations can differ between active and sham tES, and coil noise and muscle activation make TMS sham challenging. Trials should ask participants and therapists to guess allocation and report the success of blinding. For home-based stimulation, remote supervision may improve access, but only after reliable device locking, identity verification, skin checks, protocol adherence, emergency escalation, and equitable digital access have been demonstrated.

## 8 Factors contributing to heterogeneous outcomes

One of the most consistent observations in the reported literature is the marked variability in treatment effects across studies. This variability is unlikely to reflect a single methodological weakness. Rather, it arises from the interaction of biological, methodological, and analytical factors that influence both the neural effects of stimulation and their behavioural expression. Understanding these sources of heterogeneity is now more important than repeatedly asking whether neuromodulation “works,” because future progress will depend on identifying the conditions under which it is most likely to be effective.

### 8.1 Lesion-aware modelling and electric-field shaping

Biological variability probably represents the greatest challenge for neuromodulation research in aphasia. The label “post-stroke aphasia” encompasses patients with markedly different lesion topography, tissue viability, network disconnection, premorbid language organization, vascular burden, and recovery trajectories (Norise et al., 2017). Consequently, two individuals with comparable aphasia severity may exhibit profoundly different patterns of residual network organization and experience markedly different intracranial electric fields despite receiving the same stimulation protocol. These differences are likely to contribute substantially to treatment-response variability and cannot be captured by aphasia syndrome classification alone. Likewise, baseline severity should not be regarded solely as a prognostic factor but modelled appropriately, as it is intrinsically related to the potential for behavioural improvement (Harvey and Hamilton, 2022).

Importantly, anatomical variability can also alter the physical delivery of electrical stimulation. Stroke lesions introduce tissue compartments with electrical properties that differ from those of healthy brain tissue and may be accompanied by cortical atrophy, enlarged cerebrospinal-fluid (CSF) spaces, ventricular enlargement, and changes in tissue boundaries. Because current distribution depends on tissue conductivity and geometry, these alterations can modify the pathways through which current flows and consequently change both the magnitude and spatial distribution of the intracranial electric field (Bikson et al., 2012; Datta et al., 2012; Galletta et al., 2015; Saturnino et al., 2019). Indeed, computational modelling in a patient with post-stroke aphasia demonstrated that different electrode montages produced markedly different cortical electric-field distributions, highlighting the importance of considering the individual anatomy when interpreting the effects of tDCS (Galletta et al., 2015). In particular, CSF has substantially higher conductivity than brain tissue and can therefore act as a preferential pathway for current, potentially redistributing stimulation away from the intended cortical target. Stroke-related tissue loss and lesion location may consequently produce substantial inter-individual differences in electric-field strength and orientation even when identical scalp electrode montages and stimulation intensities are used.

This issue may be especially relevant when stimulation targets regions outside the canonical perisylvian language network, such as the cerebellum, supplementary motor areas, or other distributed nodes of the language network. In these cases, the relationship between electrode position and the neural tissue exposed to the strongest electric field may be less intuitive and more strongly influenced by individual anatomy. Computational approaches on finite-element modelling implemented in tools such as SimNIBS can incorporate individual anatomical information to estimate the electric-field distribution produced by different montages (Saturnino et al., 2019). Patient-specific modelling may therefore help determine whether a nominally identical montage actually produces comparable stimulation across patients and whether the intended neural target is effectively engaged.

These considerations suggest that personalization should not be restricted to selecting the cortical target according to lesion location or functional connectivity. It should also consider the individual electric field generated by the selected montage. Future studies should therefore combine lesion-aware head modelling with structural and functional connectivity measures to determine whether individualized electric-field optimization improves target engagement and, ultimately, clinical outcomes. Importantly, computational modelling should be treated as a means of improving the biological interpretability and reproducibility of stimulation rather than as evidence of clinical efficacy in itself. Prospective studies will be needed to establish whether model-guided electric-field shaping provides an incremental clinical benefit over conventional montage selection.

### 8.2 Intervention heterogeneity

A second source of variability arises from the interventions themselves. Across studies, stimulation protocols differ in target selection, stimulation parameters, treatment schedule, and timing relative to speech-language therapy. Equally important, behavioural interventions often differ substantially despite being described using similar terminology. For example, “anodal left IFG stimulation” may produce very different electric-field distributions depending on electrode placement and individual anatomy, while “naming therapy” may range from simple repeated picture naming to theoretically motivated lexical-semantic treatments. Consequently, apparent discrepancies between studies may reflect differences in the interventions being delivered rather than genuine inconsistencies in the biological effects of neuromodulation. Improving reproducibility will therefore require greater methodological harmonisation together with transparent reporting of both stimulation protocols and behavioural therapy.

### 8.3 Outcome and analytic heterogeneity

Outcome selection is another source of inconsistency. Studies vary widely in their choice of language measures, primary endpoints, follow-up intervals, and statistical approaches, making direct comparison difficult even when interventions are similar. Improvements on trained items, although sensitive to treatment effects, do not necessarily translate into generalised language recovery or functional communication. Future trials should therefore adopt clinically meaningful primary outcomes while systematically evaluating generalisation, participation, quality of life, and long-term maintenance. The development of core outcome sets would substantially improve comparability across studies.

### 8.4 Publication and sample-dependence problems

Finally, the published literature may overestimate treatment efficacy because small positive studies are more likely to be reported than negative ones. In addition, several influential publications derive from overlapping patient cohorts and therefore should not be interpreted as independent replications. Future systematic reviews should account for sample overlap, trial registration, and prespecified outcomes. Equally important, null findings should be regarded as informative because they help define the limits of neuromodulation rather than simply its successes.

## 9 Towards personalised neuromodulation

The evidence reviewed here suggests that the field is gradually moving away from the search for universally effective stimulation protocols towards more individualised neuromodulation strategies. Rather than asking which protocol works best on average, an increasingly important question is which intervention is most likely to benefit a given patient, based on their lesion characteristics, residual network architecture, behavioural profile, and treatment-related biomarkers. Recent aphasia-specific evidence supports this direction. Ren et al. (2023), for example, used resting-state functional connectivity to identify an individualized superior frontal gyrus target for theta-burst stimulation and reported greater improvements in WAB-R Aphasia Quotient in both active stimulation groups compared with sham. This study was also highlighted as an exemplar of personalized targeting in the recent meta-analysis by Wei et al. (2026). Similarly, the functionally guided rTMS trial of Li et al. (2026), together with the tract-based findings reported by Ilardi et al. (2026) and Johnson et al. (2026), further suggests that individual differences in structural and functional network organisation may provide clinically relevant information for treatment selection.

However, the identification of an individualized target does not establish that personalization improves clinical outcomes. Clinically responsible personalization requires prospective evidence that biomarker-guided treatment selection provides a meaningful advantage over conventional targeting or other clinically available approaches. This will require adequately powered randomized controlled trials in which the personalization strategy is defined *a priori*, tested prospectively, and directly compared with a standardized treatment approach. Importantly, such studies should establish not only whether individualized biomarkers predict treatment response, but whether their use provides incremental clinical benefit beyond information that can already be obtained from behavioural assessment, lesion characteristics, or other routinely available clinical measures. The reliability and reproducibility of the proposed biomarkers, their generalizability across patients and centres, and the feasibility and cost of implementing individualized targeting in routine clinical settings will also need to be established.

This transition therefore requires moving from *post-hoc* identification of predictors of treatment response towards prospectively validated treatment-selection algorithms. Future studies should determine which combination of lesion anatomy, structural and functional connectivity, computational electric-field modelling, and behavioural phenotyping provides sufficient incremental predictive value to guide treatment selection. Evidence from other neurological and neuropsychiatric conditions is beginning to support this framework, including head-to-head comparisons of connectivity-based versus conventional scalp-based targeting (Taylor et al., 2026), network-based approaches to identifying syndrome-specific stimulation targets (Siddiqi et al., 2020), and functional-connectivity-guided targeting within accelerated stimulation protocols (Cole et al., 2020). For post-stroke aphasia, the next step should therefore be to test reproducible and prospectively defined personalization strategies in randomized controlled trials, while determining which patient characteristics and neurobiological markers provide the greatest added value for predicting and optimizing treatment response.

## 10 Clinical implications

For current practice, four principles are defensible. First, neuromodulation should be considered an adjunct rather than a replacement for speech-language therapy. Second, neither hemisphere nor polarity should be selected from diagnosis alone. Lesion anatomy, recovery phase, residual network function, and the therapy task matter. Third, the expected benefit should be framed modestly: pooled effects are generally small to moderate and may be confined to language domains. Fourth, efficacy should be judged by more than improvement on practised items.

Among available techniques, rTMS currently has the strongest evidence base, whereas conventional tDCS appears easier to implement but yields smaller, more variable effects. Other approaches—including cerebellar stimulation, tsDCS, tACS, and tPBM—remain investigational. Importantly, treatment decisions should not rely solely on aphasia subtype or hemisphere of stimulation. Lesion characteristics, recovery stage, residual network integrity, and the behavioural intervention with which stimulation is paired are likely to be equally important determinants of treatment response. Finally, clinical benefit should be judged not only by improvements on trained language tasks but also by gains in functional communication and everyday participation.

## 11 Conclusion

Twenty-five years of research have established that neuromodulation can enhance language recovery under specific conditions. The central challenge is no longer to determine whether neuromodulation works, but to understand for whom, when, and through which mechanisms it is most effective. Addressing this challenge will require moving beyond standardised protocols towards biologically informed, network-based, and patient-specific treatment strategies integrated with high-quality speech-language therapy.
